# Author Correction: Nuclear genetic control of mtDNA copy number and heteroplasmy in humans

**DOI:** 10.1038/s41586-024-07364-6

**Published:** 2024-06-03

**Authors:** Rahul Gupta, Masahiro Kanai, Timothy J. Durham, Kristin Tsuo, Jason G. McCoy, Anna V. Kotrys, Wei Zhou, Patrick F. Chinnery, Konrad J. Karczewski, Sarah E. Calvo, Benjamin M. Neale, Vamsi K. Mootha

**Affiliations:** 1 Howard Hughes Medical Institute and Department of Molecular Biology, Massachusetts General Hospital, Boston, MA USA; 2 Broad Institute of MIT and Harvard, Cambridge, MA USA; 3 Analytic and Translational Genetics Unit, Center for Genomic Medicine, Massachusetts General Hospital, Boston, MA USA; 4 Department of Clinical Neurosciences, University of Cambridge, Cambridge, UK; 5 MRC Mitochondrial Biology Unit, University of Cambridge, Cambridge, UK; 6 Department of Systems Biology, Harvard Medical School, Boston, MA USA

**Keywords:** Population genetics, Mitochondrial genome, Bioenergetics, Genome-wide association studies

Correction to: *Nature* 10.1038/s41586-023-06426-5Published online 16 August 2023

When performing a genome-wide association study (GWAS), the choice of which allele (reference vs. alternate) is considered the “effect allele” is arbitrary. In our computations, we chose the reference allele. For a small subset of our results, the effect sizes were erroneously attributed to the alternate allele. The conclusions in our paper are not impacted, as we only use GWAS results to make inferences regarding the presence/absence of associations (i.e., using *P* values).

In terms of text, we have corrected one sentence in the main text. In the second sentence of the second paragraph of the subsection “Blood composition influences bulk mtCN” originally reading “…Mendelian randomization showed that effect size loci at GWS for neutrophil count were strongly positively correlated with corresponding…”, the text “positively” should have read “negatively”. This sentence also contains a typographical error that was corrected: the text “effect size loci” should read “effect sizes of loci”.

In the original Figure 1e, we displayed fine-mapped, protein-altering variants with PIP > 0.1, intentionally depicting the impact of the alternate rather than the reference allele. Though the allele frequencies were correctly reported, the effect sizes incorrectly corresponded to the effect of the reference allele. This has now been corrected by reversing the signs listed in the “Effect” column. (In the revised version, “–0.106; –0.166; –0.021; –0.109; –0.035” now have minus signs and all other values are now positive, flipped from the original version).

Our Mendelian randomization (MR) analyses relied on summary statistics from our GWAS as well as those computed previously as part of the Pan UKBB Project^1^. These previously computed GWAS summary statistics used the alternate allele as effect, while we used the reference allele. This resulted in the directionality of the *y* axis of MR plots being reversed. Since the magnitudes of the effect sizes are correct, *P* values of the test of association between effect sizes are correct and no conclusions were impacted. We have now corrected our MR plots (Figs. 1g, 1h; Extended Data Figs. 4g, 4h, 6) by correctly matching the effect alleles. (In all corrected panels, only the sign of the *y* coordinates of points are changed, now reversed, resulting in the red and black traces being flipped from the original versions).

No data in the Supplementary Tables were impacted. However, we have updated the legends of Supplementary Tables 2 and 3 for maximum clarity: these now state “Effect size estimates [and frequencies] are reported with respect to the reference (effect) allele” where the bracketed text is found only in Supplementary Table 2 where it is relevant.

This also impacted all GWAS summary statistics deposited in GWAS Catalog, which previously listed the wrong allele (the alternate allele) as the effect allele. These have now been corrected such that the reference allele is listed as the effect allele and alternate is listed as other allele. No other columns are impacted. All links to GWAS Catalog files as well as accession numbers remain unchanged. No changes to summary statistics deposited to GCP or the AllofUs workspace were required.

Separate from the above issue, we make two additional minor corrections to the text. First, in the main text, in the first sentence of the second paragraph of the subsection “Blood composition influences bulk mtCN” originally reading “indeed, elevations in inflammatory blood cell indices predict elevated risk for 26 of 29 tested diseases in UKB,” the text “26” should instead have read “25”. In the legend to Extended Data Fig. 4b, “Mean mtCN_corr_” was written and has been corrected to “Mean mtCN_adj_”. In the Supplementary Note, we noticed that citation numbering was off by one digit and in a few instances pointers to main figures were incorrect; these have been corrected. All changes to the text, figures and Supplementary information are reflected in the PDF and HTML versions of the article.
