# Supplementary material for: Nuclear genetic control of mtDNA copy number and heteroplasmy in humans
Source: Nature. 2023 Aug 16;620(7975):839–48. doi: 10.1038/s41586-023-06426-5 (PMC10447254; doi:10.1038/s41586-023-06426-5)
Supplement: Supplementary file 1 — These notes clarify methodology, justification for associated analytical choices and additional supporting interpretation for data presented in the paper. Includes Supplementary Notes 1–7, Figs. 1–7 and references 90–92. [file 41586_2023_6426_MOESM1_ESM.pdf]

nature portfolio

https://doi.org/10.1038/s41586-023-06426-5

Supplementary information

Nuclear genetic control of mtDNA copy number and heteroplasmy in humans

In the format provided by the authors and unedited

SUPPLEMENTARY NOTES

Supplementary note 1 – full description of mtSwirl pipeline

Upon ingestion of the aligned whole genome read file, we use GATK v4.2.6.0 PrintReads to quickly subset from the CRAM / BAM file to a much smaller file containing reads mapping to either the mtDNA, any intervals provided as reference NUMTs, or any other unmapped reads. Any paired-end reads which span the boundary of a reference NUMT interval are removed; we extend each NUMT interval by 500 bases prior to input to avoid any significant loss of coverage in the body of the interval.

We use these reads to perform the first round of variant calling in the NUMT regions and on the mtDNA, implementing filters to ensure only the highest quality variant calls are considered for self-reference construction. For nuclear DNA variant calls we use GATK best practices for single-sample variant calling with HaplotypeCaller. For SNVs we remove calls with QD < 2.0, QUAL < 30, SOR > 3.0, FS > 60.0, MQ < 40.0, MQRankSum < -12.5, ReadPosRankSum < -9.0, and a read depth of < 10. For INDELs we remove calls with QD < 2.0, QUAL < 30.0, FS > 200.0, SOR > 10.0, ReadPosRankSum < -20.0, and a read depth of < 10. For the mtDNA we run Mutect2 in mitochondria mode using settings as described previously[21], removing any variants flagged by FilterMutectCalls or overlapping regions previously described as artifact-prone (300-302; 309-310; 315-316; 3106-3107; 16181-16182). We use Haplochecker[58] to estimate mitochondrial contamination and remove mtDNA variant calls potentially influenced by contaminated reads using FilterMutectCalls.

First round variant calls are next used to construct a self-reference sequence for each sample. Specifically, we filter all high-quality variant calls to homoplasmies (HL > 0.95, mtDNA) or homozygous alternate (NUMT regions) calls. In rare instances, we observed homoplasmic variant calls on the mtDNA that were overlapping (e.g., a homoplasmic chrM:513:GCACA,G and a homoplasmic chrM:513:G,C) – we remove overlapping variants and flag these samples. In NUMT regions, rare instances of overlapping variants or variants overlapping interval boundaries are skipped. The consensus sequence is constructed with bcftools v1.16 consensus[56] using the final high quality homoplasmic and homozygous variants list for the mtDNA and NUMT regions respectively. The pipeline also produces a shifted version of the mtDNA consensus sequence as done previously[21] to improve read mapping to the ends of the circular chromosome.

To facilitate read mapping, we reproduce several reference files in self-reference coordinates including the coordinates of the mtDNA control region, the length of the mtDNA, any blacklisted intervals, and chain files to allow for coordinate mapping between the self-reference genome and GRCh38. Liftover of coordinates was performed using UCSC liftOver tools (https://github.com/ucscGenomeBrowser/kent). We also produce a VCF containing the set of mtDNA high quality homoplasmies used for consensus construction in self-reference coordinates – these are used to produce "force-calls" using Mutect2.

For the second round of variant calling, we produce an unmapped SAM file and then use bwa mem (with parameters -K 100000000 -p -v3) to map reads onto the self-reference and shifted self-reference sequences containing the mtDNA and NUMT regions. Picard MarkDuplicates and SortSam are subsequently used to pre-process the set of mapped reads prior to extracting only reads mapping to chrM. Mutect2 is then used in mitochondria mode to call variants on the mtDNA and shifted mtDNA using the same parameters as in the first round of variant calling, including the "force-call" variants to ensure that variant call data is obtained from sites that are homoplasmic relative to GRCh38 and incorporated into the consensus sequence. Calls from the non-coding region as obtained from the shifted mtDNA are merged with calls elsewhere on the mtDNA and artifactual variant calls, including those likely influenced by contamination (as estimated during the first round of variant calling), are filtered using FilterMutectCalls.

We note that for 1000G samples, which have very high mtDNA coverage likely due to EBV transformation as previously observed, many of the indel variants of interest had the "strand bias" filter from FilterMutectCalls. On inspection, these filtered calls appeared to retain maternal transmission (**Supplementary note 6**), and on inspection of the alignment these regions did not appear substantially different from alignments from different datasets. Thus, we allowed variant calls with the "strand bias" filter for the 1000G samples only.

The final step of the pipeline involves returning variant calls and per-base coverage estimates to GRCh38 for use in population-scale analyses. We do this via a two-step approach: first we use Picard LiftoverVcf to move "simple" variants back to GRCh38. Many variants fail this step – all force-called variants, for example, are former heteroplasmies in GRCh38 that were incorporated into the self-reference and thus have a reference allele that does not match the GRCh38 sequence. We developed a custom Liftover pipeline which systematically handles all major cases of Liftover failure, including reference allele mismatch, variants present within homoplasmic insertions relative to GRCh38, deletions spanning sites which differ between the consensus sequence and GRCh38, insertions which share a position with a homoplasmic deletion relative to GRCh38. Across >4,000,000 variant calls across 199,919 samples in UKB, we observed only 73 variants across 64 samples that were "failed" by both Picard LiftoverVcf and our custom Liftover pipeline. For per-base coverage, we use kent LiftOverTools to lift self-reference coverage estimates back to GRCh38. In instances where deletions relative to GRCh38 were incorporated into the self-reference, the allele depth of the reference allele of the homoplasmic variant call (relative to GRCh38) of these deletions is used as the per-base coverage in these gaps.

Supplementary note 2 – analysis of technical and biological covariates in mtDNA phenotypes

We closely analyzed mtDNA phenotypes for associations with technical and biological covariates, with the former including assessment center, time of day of blood draw, fasting time, month of year, and assessment date, and the latter including blood cell composition and mtDNA population structure (i.e., haplogroup).

We started by assessing the relationship between mtDNA phenotypes and blood cell type percentages and mean blood cell volumes (**Methods**). All analyses were performed with

log(mtCN) as the response variable. We observed strong linear relationships between log(mtCN) and most tested blood phenotypes (**Extended data figure 3c**), with a joint linear model of tested blood phenotypes explaining close to 23% of the observed variance in log(mtCN) (**Figure 1a**). In contrast, on testing heteroplasmies with a joint linear model of blood cell phenotypes, we observed only 3 heteroplasmies with evidence of having at least one nonzero linear model coefficient (F-test $p < 0.05$ after Bonferroni correction; **Extended data figure 11a**). We ran GWAS of these three case-only heteroplasmies (chrM:16093:T,C, chrM:16182:A,ACC, chrM:16183:A,C) with and without blood cell phenotype correction and observed very little visible difference in spectrum of genome-wide significant hits (**Extended data figure 11c-11e**). Based on these results, we implemented corrections for blood cell phenotypes in analyses of mtCN only.

We next investigated time of day of blood draw, fasting time, assessment date, and assessment center as technical covariates (see **Methods** for model specification). We found that UKB samples processed in 2006 (N = 51) showed a significantly different average mtCN compared to other periods (**Extended data figure 3e**). Given the small sample size of this period, the fact that this was a pilot, and the different average mtCN, we exclude samples collected in 2006 from all subsequent analyses. For subsequent analyses of technical variables on mtCN, we pre-correct mtCN by obtaining residuals from the model: $\log mtCN \sim blood\ cell\ variables$. Blood-adjusted mtCN showed seasonal variability (**Extended data figure 3f, 3g**), differences across fasting time (**Extended data figure 3h**), differences as a function of the time of day at which blood was drawn (**Extended data figure 3i**), and evidence of heterogeneity across assessment center (**Extended data figure 3j**). A model consisting of blood draw time, fasting time, assessment date, and assessment center explained ~3% of the variation in $mtCN_{raw}$ (**Figure 1a**) with significant evidence in support of non-zero coefficients (F-test p-value < 2.2e-16). Similar patterns were not observed for case-only heteroplasmy measures across all tested variants – the same technical covariate model showed variable R^2 estimates across the case-only mtDNA heteroplasmies (**Extended data figure 11a**), and failed to show evidence in support of any coefficients significantly different from zero in all cases (F-test p-value > 0.05 after Bonferroni correction) other than the variant chrM:567:A,ACCCCCC. Adjusted R^2 estimates showed greatly reduced magnitude for heteroplasmy traits while remaining stable around ~0.03 for mtCN (**Extended data figure 11a**). We conducted case-only GWAS (**Methods**) for chrM:567:A,ACCCCCC with and without correction for technical covariates and found minimal-to-no changes in the prevailing genome-wide architecture with correction (**Extended data figure 11b**). Based on these results, we implemented corrections for these technical covariates in analyses of mtCN only.

We assessed the impact of mtDNA population structure via haplogroup and homoplasmic mtDNA variation on $mtCN_{adj}$. We observed that top-level haplogroup showed very small but significant associations with $mtCN_{adj}$ (**Extended data figure 4a**), with top-level haplogroup explaining < 0.5% of the variance in $mtCN_{adj}$. As haplogroups form a nested tree structure of ancestral variation, we next asked how much $mtCN_{adj}$ varied between "level 2" haplogroups within each top-level haplogroup. We found very little evidence of mean differences in log(mtCN) across level 2 haplogroup within each top-level haplogroup (**Extended data figure 4b**). As a sensitivity analysis, we repeated the $mtCN_{adj}$ GWAS including top-level haplogroup indicator variables as covariates

in the GWAS model and observed no observable change to the genome-wide architecture (**Figure 1d**, **Extended data figure 4e**). Thus, we do not correct mtCN for haplogroup in our analyses.

Finally, we examined mtDNA population structure correlations with tested mtDNA heteroplasmies. In contrast with $mtCN_{adj}$, we observed many significant associations of top-level haplogroup on case-only heteroplasmy levels (**Extended data figure 11f-11g**) and thus include top-level haplogroup indicator variables in all heteroplasmy GWAS (case-only and case/control, **Methods**). Given the tree structure of haplogroups, we wondered if "deeper" haplogroups also contributed substantial variance to case-only heteroplasmy levels. To avoid inclusion of a prohibitively large set of haplogroup indicator variables, we instead computed the first 30 principal components (PCs) using all QC-pass homoplasmies with MAF > 0.001 in UKB (**Methods**). We found strong separation of samples belonging to different top-level haplogroup (**Extended data figure 8b**) as a function of the top PCs, and observed that top 30 PCs have substantial predictive power in assigning samples within each top-level haplogroup to the appropriate "level 2" haplogroup (**Extended data figure 11h**), indicating that the top 30 mtDNA PCs can effectively replace inclusion of dummy variables for top-level haplogroup while also accounting for deeper levels of haplogroup structure. To test the influence of deeper haplogroup structure on the identified genome-wide architecture of case-only heteroplasmy, we repeated GWAS for 7 representative mtDNA heteroplasmies (chrM:302:A,AC; chrM:302:A,ACC; chrM:302:A,ACCC; chrM:567:A,ACCCCCC; chrM:955:A,ACC; chrM:16179:CA,C; chrM:16183:A,C) now including 30 mtDNA PCs as covariates instead of top-level haplogroup in the GWAS model. We found no evidence of changes in estimated effect sizes for lead SNPs when using 30 mtDNA PCs instead of top-level haplogroup (**Extended data figure 11i**). Thus, as including mtDNA PCs did not appear to meaningfully influence genetic associations, we include only top-level haplogroup dummy variables in the GWAS model for all heteroplasmy traits.

The final mtCN correction model from which residuals were obtained was:

$$\begin{aligned}\log mtCN \sim\ & ns(blood\ draw\ time, 5) + assessment\ center + fasting\ time \\ & + ns(assessment\ date, SEASONAL\ KNOTS) + month\ of\ assessment \\ & + blood\ cell\ variables\end{aligned}$$

where the blood cell variables used were hematocrit percentage, platelet crit, monocyte percentage, basophil percentage, eosinophil percentage, neutrophil percentage, reticulocyte percentage, high light scatter reticulocyte percentage, immature reticulocyte fraction, mean corpuscular volume, mean reticulocyte volume, mean sphered cell volume, and mean platelet thrombocyte volume.

For our heteroplasmy phenotypes, we do not apply any corrections and include the usual covariates as well as top-level haplogroup as covariates in the GWAS model (**Methods**). Although we believe our results indicate that heteroplasmy is more robust to technical variation than mtCN, we do have significantly fewer measurements of the common heteroplasmies than mtCN, reducing our power to resolve very subtle influences of these covariates on variant heteroplasmy.

Supplementary note 3 – use of AllofUs data for mtCN and heteroplasmy analyses

We quantified all mtDNA phenotypes in both UKB and AoU (**Methods**). Through extensive testing in UKB, we found that technical and biological covariates (e.g., blood cell composition at time of blood draw, date of blood draw, assessment center) had profound impacts on mtCN measurements (**Supplementary note 2, Extended data figures 3, 4**) with covariates collectively explaining over 26% of the variance in mtCN (**Figure 1a**). Blood cell composition at the time of blood sampling has a particularly large impact on both phenotypic correlations (**Figure 1f, Extended data figure 3k, 3l**) and genetic locus discovery (**Extended data figure 4f**) in UKB. Unfortunately, these variables were unavailable in AoU. We observed a suspicious bimodal distribution of mtDNA coverage and mtCN in AoU (**Extended data figure 3a, 3b**); corrections for assessment state and sequencing center did not ameliorate this pattern. The two modes show vastly different mtCN estimates (~80 per cell versus ~200 per cell), which we believe is too large to be driven by typical biological differences in the population and is more likely to be driven by technical factors (e.g., differences in the fraction of blood used, such as buffy coat versus whole blood, or differences in DNA extraction method). Information on these factors in AoU was unavailable. Thus, we restricted our analysis of mtCN to UKB only to avoid adding bias from uncorrected analyses in AoU.

Importantly, our analysis in UKB showed that heteroplasmy measurements were quite robust to these technical variables (**Supplementary note 2**), with haplogroup being the major covariate associated with heteroplasmy levels (**Supplementary note 2**). We do not include technical or blood composition covariates in our heteroplasmy GWAS or in phenotypic correlation analyses as we observed that very few heteroplasmy traits showed significant associations with any tested covariate (**Extended data figure 11a**). We performed sensitivity analyses including these covariates in the GWAS model for the four heteroplasmy traits that showed a significant phenotypic association with technical and/or blood composition traits and found no noticeable change in the identified genetic architecture (**Extended data figure 11b-11e**). Thus, we proceeded with use of AoU mtDNA heteroplasmy calls as replication for UKB, including inferred top-level haplogroup as a covariate along with age, sex, age^2, interactions, and PCs in AoU genetic analyses (**Methods**). Importantly, this is the same GWAS model used in UKB. Encouragingly, we observed excellent replication of UKB cross-ancestry meta-analysis lead SNP effect sizes in the AoU cross-ancestry meta-analysis (**Extended data figure 10c**) and even in GWAS including individual ancestry groups in AoU (**Extended data figure 10d**), with some effect size attenuation seen consistent with Winner's curse.

Supplementary note 4 – improvement of residual stratification by computing per-ancestry PCs in AllofUs

As a sanity check, we extracted four well-powered, well-measured phenotypes in AoU for genetic analysis. We used a SQL query to obtain values corresponding to the measurement concept IDs: 903133, 3004501, 903115, 903118, corresponding to body height, blood glucose, systolic blood pressure, and diastolic blood pressure. We extracted the measurement date for each of these values and filtered to the latest measurement per individual for each phenotype. Because each

phenotype was potentially measured at a different date and time, we constructed an age covariate specific to each measurement, corresponding to the age of each individual at the date of measurement of each phenotype. To accomplish this we estimated age as the difference in years between the year of measurement and the year of birth, producing an "age" covariate that was specific for each sample and phenotype. We used the GWAS model as described in **Methods** with the hl.linear_regression_rows() method: age, sex, age*sex, age^2, age^2*sex, and the 16 PCs initially provided using PCA over all samples using high quality markers on chromosome 20 and 21, where all covariates incorporating age used the computed value corresponding to the estimated age of the participant at the time of measurement of each phenotype.

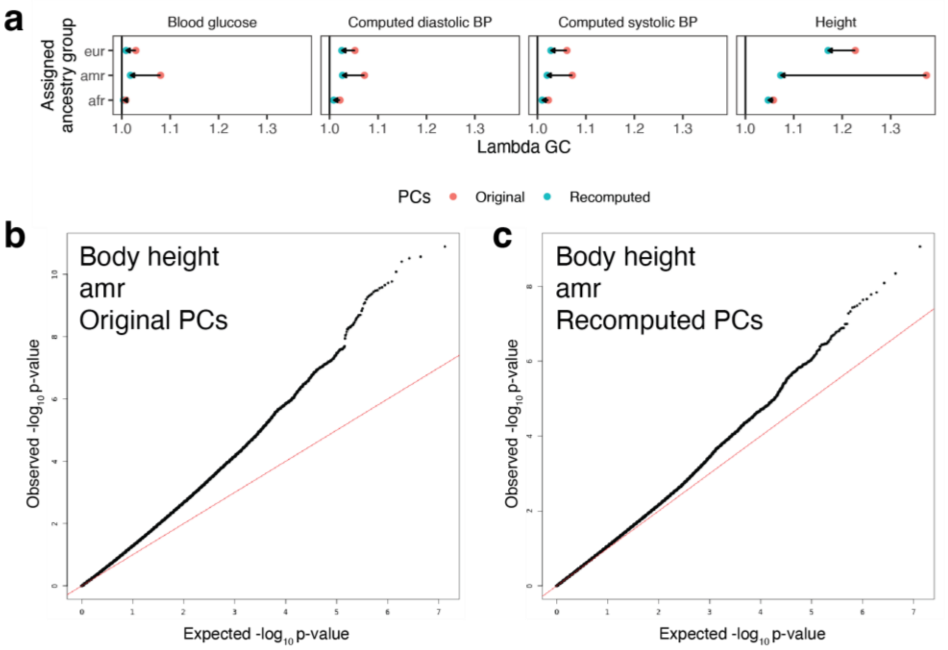

Supplemental figure S1. Improvement of population stratification with recomputed PCs. **a**. Lambda GC values obtained from GWAS summary statistics for 4 positive control phenotypes in AllofUs using original and recomputed PCs as covariates in linear regression GWAS for the three largest ancestry groups. **b**. Quantile-quantile plot of GWAS p-values for body height in the AMR group using original PCs. Red line is the y=x line. **c.** Quantile-quantile plot of GWAS p-values for body height in the AMR group using recomputed PCs.

To our surprise, we noticed evidence of population stratification for several phenotypes as elevated lambda GC (**Supplemental figure S1a**). GWAS in individuals assigned the AMR group seemed particularly susceptible, with a higher lambda GC than EUR seen for all 4 tested traits despite less than half the sample size. The most extreme example of this was for height, which showed a lambda GC of ~1.37 clearly suggestive for uncorrected stratification (**Supplemental figure S1b**).

To address this, we recomputed PCs within each assigned population group using markers distributed throughout the whole genome. We imported the AllofUs WGS variant calls in Hail, splitting multi-allelic sites and removing samples and variants failing any QC filters provided by the DRC (**Methods**). We next obtained the high-quality set of variants used for PCA in gnomAD

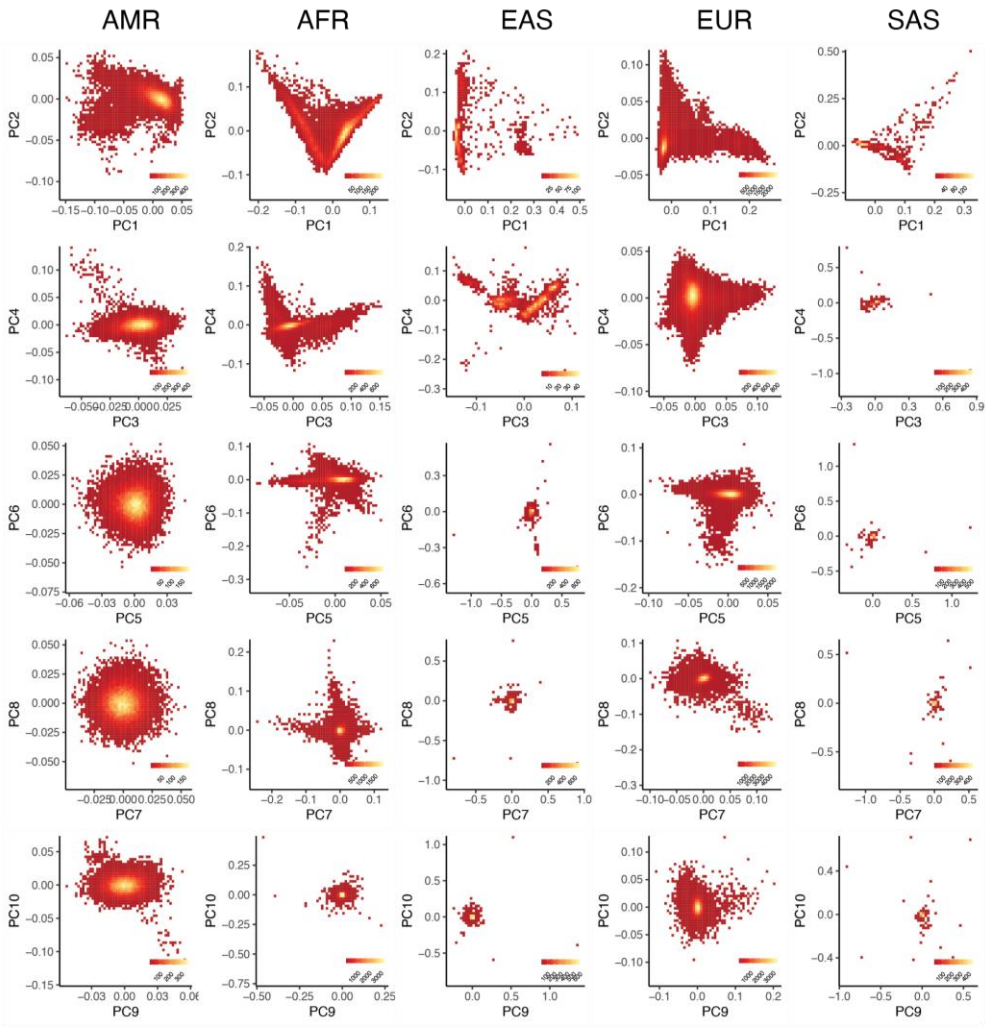

Supplemental figure S2. PCA biplots up to PC10 using PCs recomputed within each major assigned ancestry group in AllofUs. Plots are 2D histograms with per-plot legend corresponding to density scale for each plot.

v3 prior to LD-pruning. In brief, autosomal SNVs with no evidence of deviation from Hardy-Weinberg equilibrium within gnomAD (P > 1e-8), an exome call-rate of 0.99, and a gnomAD v3 minimum AC of 10 located outside of segmental duplications and low complexity regions and inside high-coverage exome intervals were obtained. See https://github.com/Nealelab/ccdg_qc/blob/master/scripts/pca_variant_filter.py for more details on the construction of the high-quality variant set. Of the 259,482 such variants identified, 258,457 variants were found in the AllofUs call-set after QC. Within each assigned ancestry, we filtered to high-quality variants with minor allele frequency > 0.001 and then ran linkage disequilibrium (LD) pruning using hl.ld_prune() with an r^2 threshold of 0.1 to identify independent high-quality markers for each ancestry group. Samples corresponding to MID were excluded given the small sample size of these samples. After filtering and pruning, 137,394 variants were included for PCA in at least 1 ancestry group. PCA was performed similarly as for UKB (**Methods**) – for each assigned ancestry group, PCs were produced using LD-pruned high-quality variants for that ancestry among unrelated individuals using the hl.hwe_normalized_pca() method, and related samples were subsequently projected into this PC space. The first 20 PCs were retained

for all samples for use as covariates for GWAS. We visualized the first 10 PCs in biplots for each of the five analyzed ancestry groups (**Supplemental figure S2**), observing generally expected population structure captured by the PCs with few examples of extreme ancestry outliers.

We reran GWAS for our four selected positive control phenotypes using all 20 newly recomputed per-population PCs as covariates and observed a substantial reduction in lambda GC relative to the provided PCs for all tested ancestry-trait pairs (**Supplemental figure S1a**). The largest decline was observed for height for the AMR group, with lambda GC falling from ~1.37 to ~1.07 (**Supplemental figure S1c**), indicating that the new PCs substantially reduce residual stratification. We use these newly recomputed PCs for all mtDNA phenotype GWAS in AoU.

Supplementary note 5 – measures taken to avoid NUMT contamination of association results

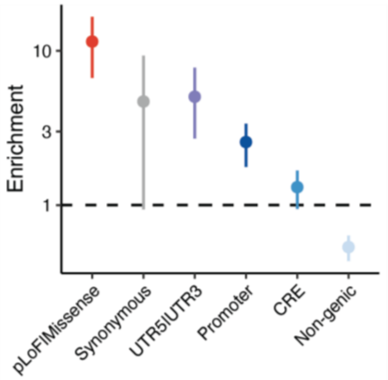

Supplemental figure S3. Enrichment of annotated genomic function for variants with maximal PIP > 0.1 across all main mtDNA analyses after fine-mapping. Dotted line corresponds to no enrichment. Error bars represent 95% CI computed via bootstrapping. Sample sizes are as follows: 15,698 (pLoF|Missense), 10,078 (Synonymous), 24,427 (UTR5|UTR3), 118,205 (Promoter), 260,970 (CRE), 1,242,767 (Non-genic).

The study of mtDNA genome sequence variation has historically been plagued by nuclear mtDNA pseudogenes called NUMTs, which can produce spurious low-heteroplasmy variant calls due to nucDNA reads from these homologous regions mis-mapping to mtDNA. Multiple lines of evidence support the robustness of our current results:

1. Our mtSwirl pipeline maps reads to a self-reference mtDNA and known NUMT regions to reduce mis-mapping (**Extended data figure 1**).
2. We exclude low-mtCN samples which are more prone to NUMT-derived variant calls (**Extended data figure 8c**)[21].
3. We have only considered heteroplasmies >5%. Variants at lower heteroplasmy are at a higher risk of being NUMT-derived.
4. We find little-to-no evidence of paternal transmission of heteroplasmies (**Figure 4c**). Paternal transmission of mtDNA variation would be expected for NUMT-derived variants[89], however all variants for which we performed genetic analysis show strong maternal transmission in UKB.

5. Our GWAS yields numerous fine-mapped nuclear loci near genes with established roles in mtDNA biology and maintenance.
6. Associated loci tend to show small credible sets after fine-mapping (**Extended data figures 5b, 10e**) with strong enrichment for functional variants across all main GWAS (**Supplemental figure S3**); this would not be expected for NUMT-based associations. NUMT-based associations are expected to purely be due to read-alignment artifact, and thus should not be correlated to variant type (e.g., missense vs. synonymous vs. noncoding).
7. Numerous sensitivity analyses yield virtually unchanged GWAS effect sizes, including correcting for overall mtCN, correcting for coverage at the mtDNA heteroplasmy site and restricting to variant calls supported by more reads than the median nucDNA coverage (**Extended data figure 11j-11m**). We expect that NUMT-driven associations would show effect sizes correlated with coverage.
8. We successfully replicated our results in an independent cohort, finding replication even across different genetic ancestry groups (**Extended data figure 10c, 10d**). Replication would be unlikely if recent polymorphic NUMTs were driving our results as recent polymorphic NUMTs are unlikely to appear across multiple nuclear genetic ancestry categories.
9. Finally, we collated an extensive database of 4,736 polymorphic and reference NUMT intervals using BLASTn and existing datasets[51,85–87] (**Methods**) and conservatively tested for LD $R^2 > 0.1$ between any SNP in a 20kb window around a NUMT and each genome-wide significant lead variant for our case-only heteroplasmy GWAS:

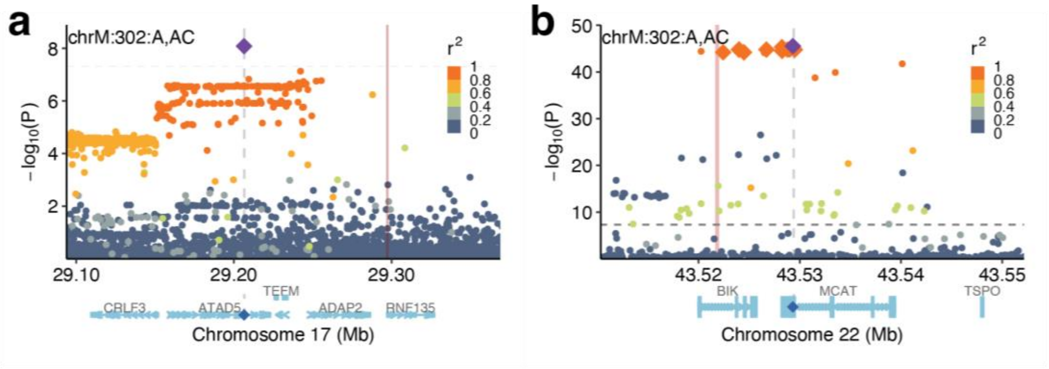

Supplemental figure S4. Locuszoom plots of genetic associations between chrM:302:A,AC and **a.** the chromosome 17 locus near TEFM and **b.** the chromosome 22 locus near MCAT. Color indicates LD R^2 to the lead variant (purple diamond). Red ribbon corresponds to polymorphic NUMT insertion point. Large sized diamonds in panel **b** correspond to variants in the 95% credible set after fine-mapping.

Among 2,961 tested reference NUMTs, we found that only the SSBP1 locus on chromosome 7 showed LD ($R^2 > 0.9$) with a NUMT region (**Extended data figure 10k**). Within this locus, while EUR individuals have high LD between *SSBP1* and the NUMT region, we observed a substantially shorter LD block among AFR individuals in AoU. Despite this reduced LD, GWAS in AFR individuals in AoU produced the *SSBP1*-proximal signal, suggesting that the NUMT is not driving the association (**Extended data figure 10k**). Furthermore, the NUMT region is homologous to chrM:2740-chrM:6623; this mtDNA region does not contain chrM:302 or any of the other

heteroplasmic sites found to associate with the SSBP1 locus, further reducing the likelihood of this nucDNA region confounding associations with our tested heteroplasmic sites. Finally, RVAS revealed an association between the levels of ultra-rare (MAF < 0.0001) missense + LoF variation within *SSBP1* and chrM:302:A,AC heteroplasmy (**Figure 5i**). Importantly, the levels of synonymous variation within *SSBP1* were not associated at any tested allele fraction in RVAS with chrM:302:A,AC heteroplasmy (**Figure 5i**). Taken together, our results provide strong evidence that the association at the *SSBP1* locus is indeed mediated via the *SSBP1* gene itself and not the nearby NUMT.

Among 1,775 polymorphic NUMTs[86,87], we find only two instances of LD between a lead SNP and any SNPs within a 20kb window around a NUMT insertion site. We note that these NUMTs would only cause spurious associations if they were in strong LD with genotyped variants, as the NUMT sequence itself is not included in the reference genome:

1. We find maximum LD R^2=0.606 between the TEFM locus and SNPs in a 20kb window around a polymorphic NUMT nearby. We do not believe this is likely to contaminate our signal for several reasons: (1) this is an ultra-rare (AF < 0.1%) NUMT found in the "oth" ancestry group rather than among the populations used in our UKB discovery analysis, (2) the mtDNA sequence inserted here is chrM:6866-7019, while the mtDNA heteroplasmy trait associated with the nearby locus is chrM:302, reducing the chances of reads from this locus altering the observed heteroplasmy at chrM:302, and (3) on examining local LD structure (**Supplemental figure S4a**) it appears that there is a sharp drop-off in LD consistent with a recombination site separating our GWAS-identified locus and the true NUMT insertion point.
2. We find that the MCAT locus is within 10kb of a nearby polymorphic NUMT (**Supplemental figure S4b**). While we cannot exclude influence of this NUMT on our GWAS signal, we believe this is not driving our signal for several reasons: (1) this locus appears significant for heteroplasmy traits, coverage discrepancy analysis, and mtCN. While the former heteroplasmy or coverage discrepancies may be sensitive to NUMT contamination, mtCN (and particularly mtCN computed using median mtDNA coverage) should be robust to polymorphic NUMT contamination; (2) the mtDNA sequence inserted is chrM:2045-2221 while the locus is associated with chrM:302, reducing the chances of reads from this locus altering the observed heteroplasmy at chrM:302; and (3) the polymorphic NUMT is ultra-rare (AF < 0.1%).

Thus, while we cannot eliminate the possibility that NUMTs may influence some of our associations, our variant calling pipeline, quality control procedure, sensitivity analysis procedure, transmission analysis, genetic analysis, fine-mapping, and LD analysis all point to our results being robust to NUMT contamination.

Supplementary note 6 – assessed indel variants are unlikely to be artifacts

Prior WGS analyses have excluded indel variants at chrM:302 and at other mtDNA sites for fear that these may arise due to sequencing artifact given their proximity to mtDNA poly-C tracts, however we believe this is unlikely to confound our results. First, we only perform genetic

association testing with common indel variants (MAC > 500) in UKB, thus excluding rare polymorphisms that are likely due to amplification artifact. Second, it is extremely improbable that this sequencing artifact could induce nuclear genetic associations nominating genes specifically involved in mtDNA maintenance and replication, as this would imply that the degree of mtDNA sequencing artifact was somehow correlated to functional variation within nuclear DNA genes (**Supplemental figure S3**). Third, we find that the chrM:302 variant appears to correlate with haplogroup, with the overall compositional spectrum of indel variants at this site differing substantially between haplogroups HV and L1 (**Figure 5d**). We do not believe it is likely that sequencing artifact could produce such profound cross-haplogroup differences. Fourth, we observe that all indel variants tested, despite being adjacent to poly-C tracts, show very strong quantitative maternal transmission in most cases, with little evidence of paternal transmission (**Extended data figure 9**); this is true specifically of the length variants at chrM:302 as well (**Figure 5b**). We do not expect sequencing artifact to behave in a manner that would induce maternal-specific transmission.

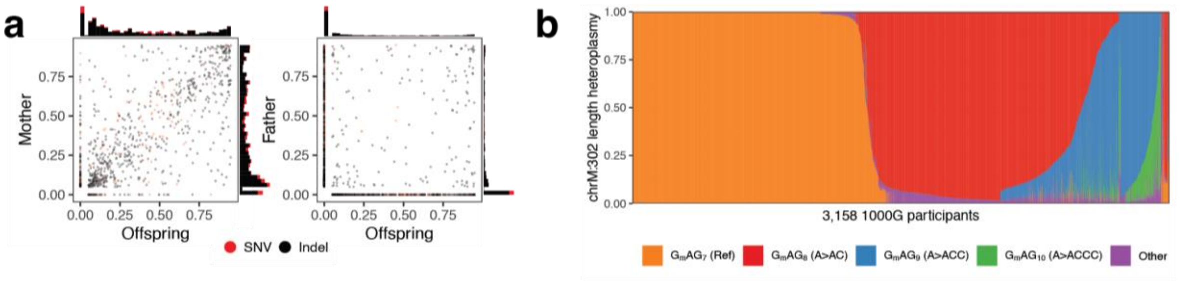

Supplemental figure S5. Spectrum of transmission and chrM:302 length heteroplasmy variation across 1000G cell lines. **a**. Transmission of 39 tested common heteroplasmies in 1000G between mother-offspring (left) and father-offspring (right) pairs across 602 families. **b**. Composition of indel variants at position chrM:302 across the 1000G cohort.

Strengthening this further, we also find a similar quantitative maternal transmission pattern among 602 trios from the 1000G cohort (**Supplemental figure S5a**). This implies that our assayed variants, including primarily indels near poly-C stretches, are stable not only through maternal-offspring mtDNA transmission but also through EBV-transformation and cell culture propagation. Again, we see no convincing evidence of paternal transmission among these cell lines. Strikingly, the detected spectrum of heteroplasmy at position chrM:302 is similar to that observed in the population (**Supplemental figure S5b, Figure 5c**), further indicating stability of these variants even through culture of patient-derived cells.

Taken together, it is very unlikely that a sequencing or PCR artifact could produce apparent quantitative maternal transmission with little detectable paternal transmission across multiple sites and datasets.

More generally, these variants have been used widely in population genetic and forensic studies, and several groups have directly quantified mixtures of length heteroplasmies at chrM:302 across several tissues[90,91] using size-based separation of PCR products and subsequent cloning and sequencing. Thus, these variants have been detected reliably in the past in the human population

using alternative methods, providing further credence to the quality of our calls and reducing the likelihood that these are universally artifactual.

Supplementary note 7 – Manhattan plots from UKB case-only GWAS with any genome-wide signals

We performed GWAS in UKB for 39 case-only heteroplasmy traits. To display these in a space-efficient manner, we show all clumped lead SNPs with $p < 5e-5$ in **Figure 4f** for traits that showed any genome-wide significant signal, and the same display for all tested heteroplasmies in **Extended data figure 10a**. In this note we enclose full Manhattan plots for each of the case-only heteroplasmy GWAS in UKB which show any genome-wide significant signal – see **Supplemental figures S6, S7**.

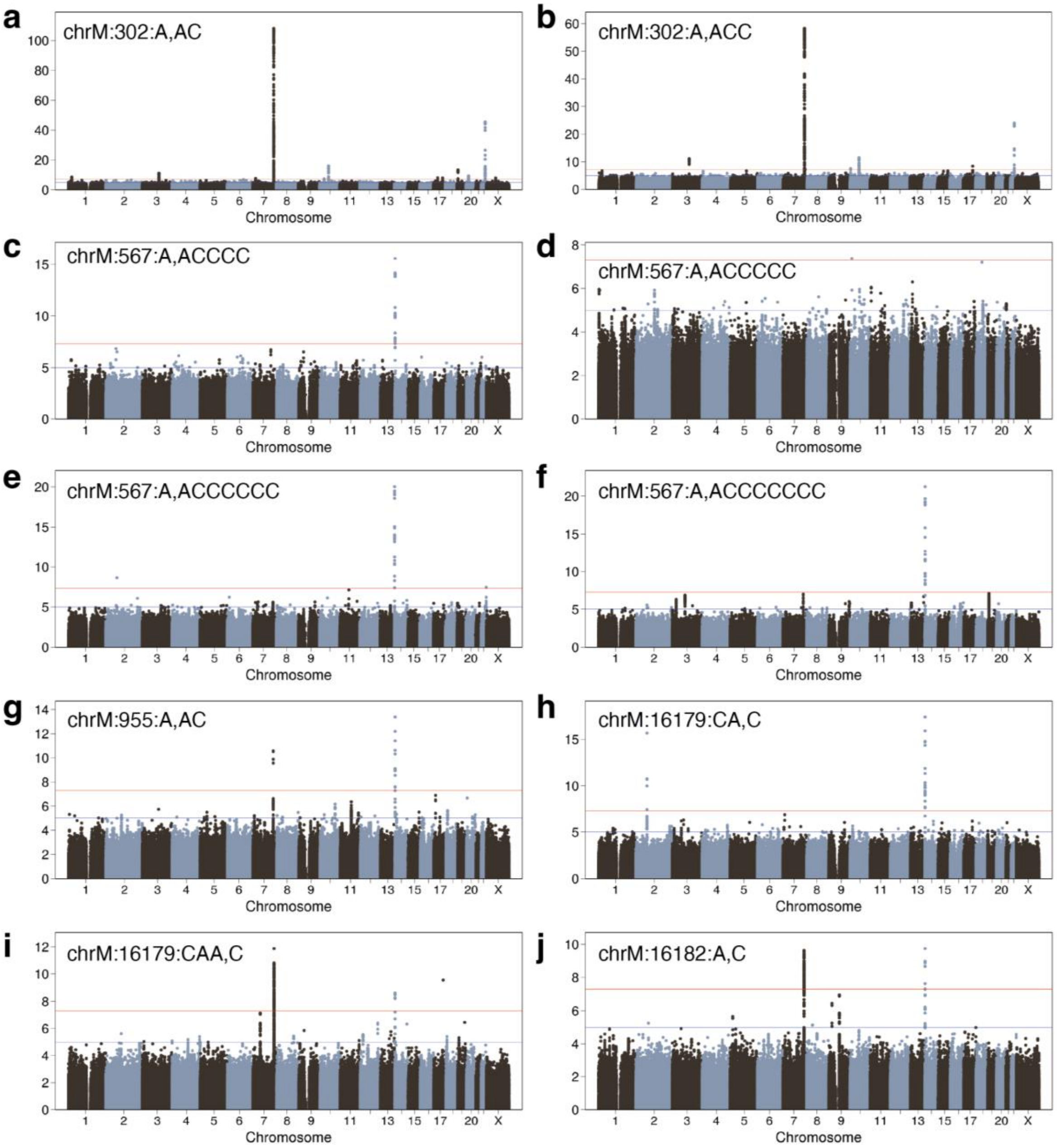

Supplemental figure S6. Manhattan plots of first 10 case-only heteroplasmies with any genome-wide significant signal detected. These correspond to the lead SNPs plotted in **Figure 4f.** Red line indicates genome-wide significance, blue line indicates “suggestive”. Y-axis is -log10(p-value), inset text is the case-only heteroplasmy tested.

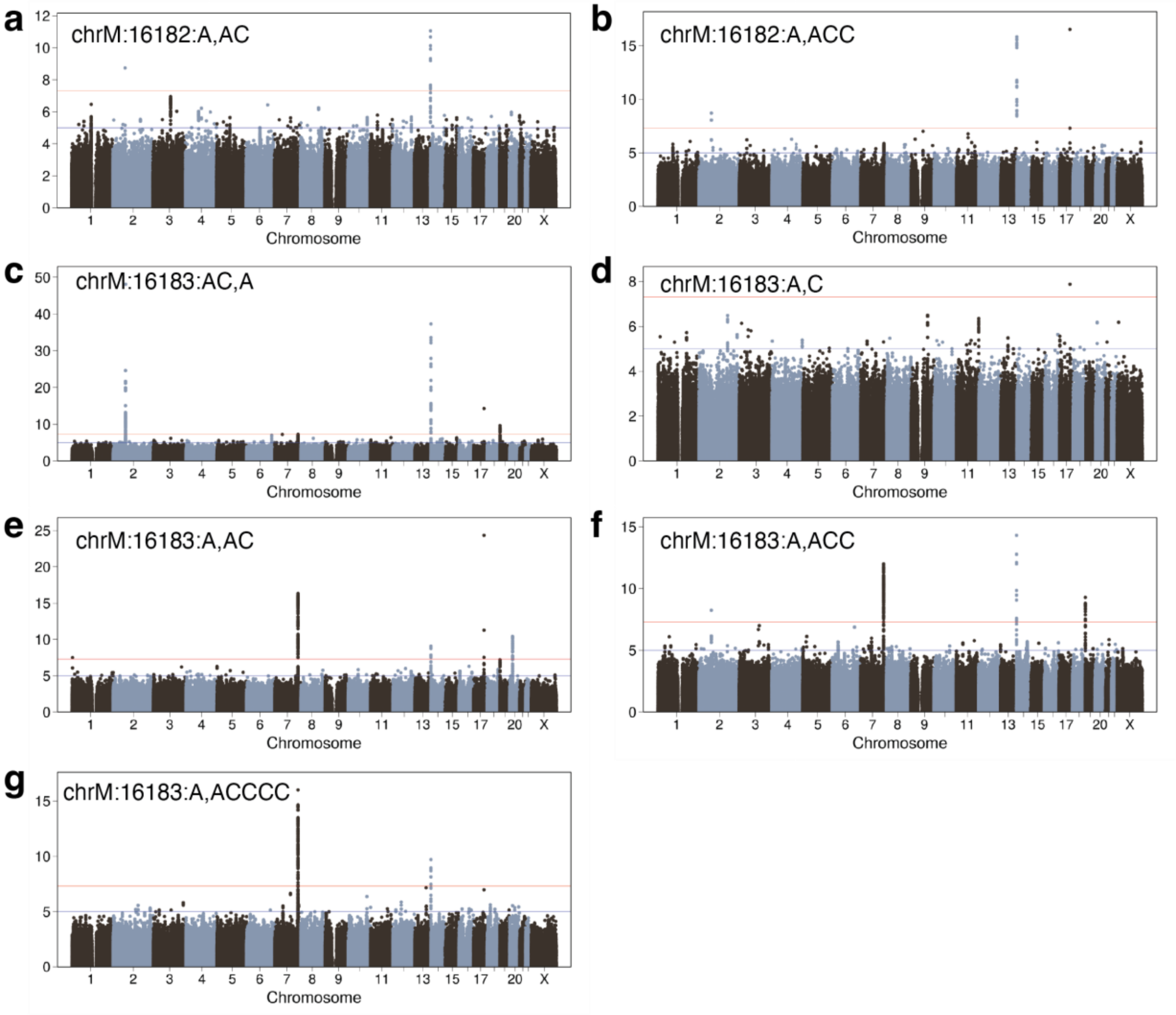

Supplemental figure S7. Manhattan plots of next seven case-only heteroplasmies with any genome-wide significant signal detected. These correspond to the lead SNPs plotted in **Figure 4f.** Red line indicates genome-wide significance, blue line indicates "suggestive". Y-axis is -log10(p-value), inset text is the case-only heteroplasmy tested.

ADDITIONAL SUPPLEMENTARY REFERENCES

89. Wei, W. *et al.* Nuclear-mitochondrial DNA segments resemble paternally inherited mitochondrial DNA in humans. *Nat Commun* **11**, 1–11 (2020).

90. Lee, H. Y. *et al.* Differential Distribution of Human Mitochondrial DNA in Somatic Tissues and Hairs. *Ann Hum Genet* **70**, 59–65 (2006).

91. Shin, M. G. *et al.* Mitochondrial DNA sequence heterogeneity in circulating normal human CD34 cells and granulocytes. *Blood* **103**, 4466–4477 (2004).
