# Supplementary material for: Nuclear genetic control of mtDNA copy number and heteroplasmy in humans
Source: Nature. 2023 Aug 16;620(7975):839–48. doi: 10.1038/s41586-023-06426-5 (PMC10447254; doi:10.1038/s41586-023-06426-5)
Supplement: Supplementary file 2 — Reporting Summary [file 41586_2023_6426_MOESM2_ESM.pdf]

nature portfolio

Corresponding author(s): Vamsi K. Mootha, Benjamin M. Neale

Last updated by author(s): Jun 21, 2023



# Reporting Summary

Nature Portfolio wishes to improve the reproducibility of the work that we publish. This form provides structure for consistency and transparency in reporting. For further information on Nature Portfolio policies, see our Editorial Policies and the Editorial Policy Checklist.

## Statistics

For all statistical analyses, confirm that the following items are present in the figure legend, table legend, main text, or Methods section.

| n/a | Confirmed | |
|---|---|---|
| ☐ | ☒ | The exact sample size (*n*) for each experimental group/condition, given as a discrete number and unit of measurement |
| ☐ | ☒ | A statement on whether measurements were taken from distinct samples or whether the same sample was measured repeatedly |
| ☐ | ☒ | The statistical test(s) used AND whether they are one- or two-sided<br>*Only common tests should be described solely by name; describe more complex techniques in the Methods section.* |
| ☐ | ☒ | A description of all covariates tested |
| ☐ | ☒ | A description of any assumptions or corrections, such as tests of normality and adjustment for multiple comparisons |
| ☐ | ☒ | A full description of the statistical parameters including central tendency (e.g. means) or other basic estimates (e.g. regression coefficient) AND variation (e.g. standard deviation) or associated estimates of uncertainty (e.g. confidence intervals) |
| ☐ | ☒ | For null hypothesis testing, the test statistic (e.g. *F*, *t*, *r*) with confidence intervals, effect sizes, degrees of freedom and *P* value noted<br>*Give P values as exact values whenever suitable.* |
| ☐ | ☒ | For Bayesian analysis, information on the choice of priors and Markov chain Monte Carlo settings |
| ☒ | ☐ | For hierarchical and complex designs, identification of the appropriate level for tests and full reporting of outcomes |
| ☐ | ☒ | Estimates of effect sizes (e.g. Cohen's *d*, Pearson's *r*), indicating how they were calculated |

*Our web collection on statistics for biologists contains articles on many of the points above.*

## Software and code

Policy information about availability of computer code

| | |
|---|---|
| Data collection | No data was collected in this study as we analyzed existing whole genome sequencing data. |
| Data analysis | We release the full WDL pipelines and associated input files for mtDNA analysis from whole genome sequencing data on GitHub (https://github.com/rahulg603/mtSwirl; DOI: 10.5281/zenodo.8067503). We also provide the code we used to run the pipeline on the UKB Research Analysis Platform, AoU, and Terra, consolidate all data, perform mtDNA sample and variant QC, and run GWAS. See Methods and the README in the GitHub repository for more information on how to use the pipeline. Several tools were used as part of mtSwirl, including GATK v4.2.6.0 (https://gatk.broadinstitute.org/), samtools v1.9 (https://github.com/samtools/samtools) and bcftools v1.16 (https://github.com/samtools/bcftools), Haplochecker 0124 https://github.com/genepi/haplocheck), R v3.1.1 (https://r-project.org), Hail v0.2.84 (https://hail.is), and UCSC kent tools source version 430 (genome-source.soe.ucsc.edu/kent.git and https://hgdownload.soe.ucsc.edu/admin/exe/linux.x86_64/).<br><br>We used several published tools and scripts to perform downstream analysis of the mtDNA callset in this study. All data wrangling, statistical analysis, and figure generation was performed using either Hail v0.2.98 (https://hail.is), python v3.7.10 (https://www.python.org), or R v4.2.1 (https://r-project.org). Parallelization of tasks in UKB was performed using Hail Batch (in Hail v0.2.98) (https://batch.hail.is) and in AoU using Cromwell v77 (https://cromwell.readthedocs.io). GWAS was performed in UKB using SAIGE v1.1.5 (https://saigegit.github.io). For scaling of UKB GWAS, a custom modification of the GWAS pipeline from the Pan UKBB pan-ancestry GWAS was implemented (https://github.com/atgu/ukbb_pan_ancestry). Linear regression GWAS was performed in AoU using Hail. We release the code used for GWAS on both UKB and AoU on GitHub (https://github.com/rahulg603/mtSwirl). mtDNA PCA was performed in R using the irlba v2.3.5.1 package (https://cran.r-project.org/web/packages/irlba/index.html). Multinomial models were trained using the nnet v7.3-17 package in R (https://cran.r-project.org/web/packages/nnet/index.html). Circos plots were made using the circlize package v0.4.15 in R (https://jokergoo.github.io/circlize_book/book/). For analysis of chrM:302 in single cell data, we used BedTools v2.29.2 (https://bedtools.readthedocs.io). LD clumping was performed using Plink v1.90 (https://www.cog-genomics.org/plink/). Finemapping was performed using FINEMAP-inf v1.3 and SuSiE-inf v1.2 (https:// |

github.com/FinucaneLab/fine-mapping-inf). eQTL data was obtained from GTEx v8 (https://gtexportal.org) and the eQTL catalogue release 4 (https://www.ebi.ac.uk/eqtl/). For replication analysis effect size comparisons, the deming v1.4 package was used in R (https://cran.r-project.org/web/packages/deming/index.html). Heritability estimates and enrichment analyses were performed using stratified LD-score regression (https://github.com/bulik/ldsc). BLASTn v2.13.0 was used as available from the NCBI (https://blast.ncbi.nlm.nih.gov/Blast.cgi). MUSCLE v3.8.31 was used for protein sequence alignment (https://drive5.com/muscle/downloads_v3.htm).

For manuscripts utilizing custom algorithms or software that are central to the research but not yet described in published literature, software must be made available to editors and reviewers. We strongly encourage code deposition in a community repository (e.g. GitHub). See the Nature Portfolio guidelines for submitting code & software for further information.

# Data

Policy information about availability of data

All manuscripts must include a data availability statement. This statement should provide the following information, where applicable:

- Accession codes, unique identifiers, or web links for publicly available datasets
- A description of any restrictions on data availability
- For clinical datasets or third party data, please ensure that the statement adheres to our policy

In terms of data processed or generated as part of this study, we provide per-population mtDNA heteroplasmic and homoplasmic allele frequencies and counts in UKB and AoU (Supplementary tables 5, 6), genetic association statistics for LD-independent lead SNPs and fine-mapped variants in UKB in addition to colocalization results (Supplementary tables 2-4), and gene-based RVAS association statistics for genes passing genome-wide significance for the Cauchy test (Supplementary table 7). All GWAS sample sizes for each genetic ancestry group, meta-analysis, and phenotype can be found in Supplementary table 1. All GWAS summary statistics from UKB cross-ancestry meta-analyses (used here in discovery analyses) have been deposited in GWAS Catalog (ID: GCP000614). Summary statistics containing all per-ancestry association statistics as well as cross-ancestry meta-analyses can be accessed via Google Cloud Platform (bucket: gs://mito-wgs-public-2023). Full GWAS summary statistics from AoU (used here as a replication cohort) have been deposited in a workspace available on AoU workbench (titled "Nuclear genetic control of mtDNA copy number and heteroplasmy in humans"; https://workbench.researchallofus.org/workspaces/aou-rw-3273c7f0/nucleargeneticcontrolofmtdnacopynumberandheteroplasmyinhumans/data). Individual level data generated as part of UKB (mtDNA copy number and mtDNA variant calls) have been returned to UKB to enable utilization of the full individual-level data by the broader scientific community via the UKB data showcase. Individual level data generated as part of AoU have been deposited in the same workspace containing summary statistics on the AoU Research Workbench. Please see our Github repository (https://github.com/rahulg603/mtSwirl) for more information on accessing these data. At the time of publication, access to the AoU workbench controlled-tier is restricted to US-based academic institutions, government entities, health care institutions, and non-profit organizations. Please also note that as of the time of publication, the only method to gain access to the AoU workspace containing the data generated here is to contact us to be added to the workspace. For information about access to the Researcher Workbench as a registered researcher, please visit https://www.researchallofus.org.

In terms of external data used in this study, we leveraged GWAS summary statistics, and ancestry-specific LD-matrices, and a curated list of 29 common, high-quality disease phenotypes generated as part of the Pan UKBB project 62. Paths for these summary statistics (https://pan.ukbb.broadinstitute.org/docs/per-phenotype-files) and LD-matrices (https://pan.ukbb.broadinstitute.org/docs/ld) can be found on the Pan UKBB project website (https://pan.ukbb.broadinstitute.org); these were accessed via Google Cloud Platform as part of this study. UKB phenotype and whole genome sequencing data can be accessed via the UKB Research Analysis Platform after completing a UKB access application (https://ukbiobank.dnanexus.com/landing). AoU phenotype and genotype data can be accessed via access to the Controlled Tier v6 on the AoU researcher workbench (https://workbench.researchallofus.org). gnomAD v3.1.2 (https://gnomad.broadinstitute.org) WGS was accessed via a custom Terra workspace (titled "gnomad_subsampled_mitopipeline_head_to_head"). High coverage WGS from 1000G was accessed using the public "1000G-high-coverage-2019" workspace in Terra. Published mtscATACseq data used for chrM:302 analysis can be obtained via approval from dbGaP. Gene-sets for enrichment analyses can be obtained using COMPARTMENTS (https://compartments.jensenlab.org) and MitoCarta 2.0 (https://www.broadinstitute.org/files/shared/metabolism/mitocarta/human.mitocarta2.0.html) as described previously 24. The GRCh37 and GRCh38 reference genomes as well as other standard reference data are available via the GATK resource bundle (https://gatk.broadinstitute.org/hc/en-us/articles/360035890811-Resource-bundle). Annotations for the baseline v1.1 and BaselineLD v2.2 models for S-LDSC as well certain other relevant reference data, including the HapMap3 SNP list, can be obtained from https://alkesgroup.broadinstitute.org/LDSCORE/. Known reference and polymorphic NUMTs were obtained from supplemental data as provided in published work 51,86–88.

# Human research participants

Policy information about studies involving human research participants and Sex and Gender in Research.

| | |
|---|---|
| Reporting on sex and gender | We did not perform new recruitment in this study and used existing cohorts and datasets. Sex was determined based on variables provided by UKB or via genetic inference in AllofUs. Sex is an important correlate with several observed phenotypes and has been used as a covariate for most analyses in this study. |
| Population characteristics | We did not perform new recruitment in this study and used existing cohorts and datasets. We did not filter on any relevant population characteristics in our analysis.<br><br>Regarding the characteristics of the datasets used in this study, briefly: UKB is a population-based cohort comprising ~500,000 individuals from ages 40-69 in the UK, recruited from sites across the country to cover a variety of socioeconomic settings and ensure an urban-rural mix (Sudlow et al. 2015 PLOS Medicine). AllofUs (AoU) is a population-based longitudinal cohort study in the US, enrolling participants age 18 or greater. AoU attempts to represent individuals otherwise underrepresented in biomedical research, and thus incorporates variables such as race, ethnic group, age, sex, gender identity, sexual orientation, disability status, income, and more in the recruiting strategy ("The 'All of Us' Research Program," 2019 NEJM). 1000G sampled participants across 26 populations around the world, assessing ~5 subgroups within each of 5 major continental populations to build a reference of genetic variation ("The 1000 Genomes Project Consortium" 2015 Nature, Byrska-Bishop et al. 2022 Cell). Phenotype data from this cohort was not used in this study. gnomAD v3 comprised opportunistically collected WGS data primarily from case-control studies of adult-onset common diseases, including cardiovascular disease, type 2 diabetes, and psychiatric disorders. Individuals with severe pediatric disease, or those with |

known first degree relatives of those with severe pediatric disease, were excluded from the cohort (Chen et al. 2022 bioRxiv, Karczewski et al. 2020 Nature). Phenotype data from this cohort was not used in this study. mtscATAC-seq data was obtained from one individual sequenced as part of recently analyzed MELAS cases (Walker et al. 2020 NEJM). Individuals were selected on the basis of a known diagnosis of carrying the m.3243A>G pathogenic variant, and not for population. All individuals from Walker et al. 2020 NEJM were male.

Neither AllofUs nor UKB select explicitly on diagnoses/treatment characteristics. Both sexes were represented in all cohorts except in mtscATAC-seq data. No filtering was performed on genotype information. The populations represented in our primary analyses are available in Supplementary table 1 and in Extended data figure 2. All datasets contained whole genome sequencing data, which was the focus of this study. More details can be found in Methods and in the relevant publications.

| | |
|---|---|
| Recruitment | We did not perform new recruitment in this study and used existing cohorts and datasets. See previously published literature for more information on the recruitment of individuals for the published datasets in this work: UK Biobank – Sudlow et al. 2015 PLOS Medicine; UK Biobank WGS – Halldorsson et al., 2022 Nature; AllofUs – “The ‘All of Us’ Research Program,” 2019 NEJM; mtscATAC-seq data – Walker et al. 2020 NEJM. |
| Ethics oversight | We did not perform new recruitment in this study and used existing cohorts and datasets. Analysis of UK Biobank data was performed under UKB Application 31063. Analysis of AllofUs data was performed under Controlled Tier authorization in the workspace “Genetic determinants of mitochondrial DNA phenotypes”. Institutional Review Board authorization of analysis of previously published single cell data was provided by Massachusetts General Hospital under protocol #2016P001517. |

Note that full information on the approval of the study protocol must also be provided in the manuscript.

# Field-specific reporting

Please select the one below that is the best fit for your research. If you are not sure, read the appropriate sections before making your selection.

☒ Life sciences ☐ Behavioural & social sciences ☐ Ecological, evolutionary & environmental sciences

For a reference copy of the document with all sections, see nature.com/documents/nr-reporting-summary-flat.pdf

# Life sciences study design

All studies must disclose on these points even when the disclosure is negative.

| | |
|---|---|
| Sample size | This was a biobank-scale analysis making use of all available whole genome sequencing samples in UK Biobank and AllofUs after quality control. Thus, no a priori sample size calculation was completed. This study comprises the largest analysis of mtDNA to date. |
| Data exclusions | Data were excluded during the study procedure for quality control or power purposes only. In brief, we excluded samples with evidence of contamination and with abnormal overlapping homoplasmy variant calls from all analysis. The former could produce incorrect mtDNA phenotype estimation or nucDNA genotype calls; the latter may indicate abnormalities in variant calling. We also excluded samples collected in the UKB pilot in 2006 as these samples had abnormal mtDNA copy number estimates. For variant analysis, we additionally excluded samples with low mtDNA copy number due to an established risk of NUMT contamination. We developed the “overlapping homoplasmy” filter and the “UKB pilot” filter during the study; the others were used previously (Laricchia et al. 2022 Genome Res) and pre-established. For genetic analyses, we restricted to samples with high confidence continental ancestry assignments in UKB (see http://pan.ukbb.broadinstitute.org) and AllofUs, and only performed GWAS for ancestry groups with enough measurements for interpretability. See Methods for more details. |
| Replication | We attempted replication for the two major components of the study: nuclear genetic analyses of (1) mtDNA copy number and (2) heteroplasmy. For (1), we obtained loci identified by the largest GWAS of mtCN previously completed by Longchamps et al. 2022; we successfully identified the vast majority of previously identified loci in our study (>85% at a stringent threshold of $p < 5e-5$). For (2), we used AoU to perform independent replication of heteroplasmy associations identified in UKB. We saw strong effect size concordance between cross-ancestry meta-analyses performed in either biobank ($R2 = 0.79$). |
| Randomization | We did not perform experimental group assignment in this study and performed genetic analysis using data from all samples that passed QC. Thus, as most of this work is population-based, this is not relevant for most of our work. In general, we extensively address potential confounders by leveraging the deep phenotyping in UK Biobank, testing and correcting for confounders such as blood cell composition, blood draw time, blood draw season, haplogroup, and others – see Methods. All genetic analyses included further corrections for population stratification by including genotype PCs computed within each genetic ancestry group, as well as age, sex, age2, age*sex, and age2*sex. Finally, for case/control disease trait associations with mtDNA phenotypes, we corrected for haplogroup, genetic ancestry group, and the aforementioned age and sex covariates. See Methods for more details. |
| Blinding | Blinding was not relevant for this study as experimental group assignment was not performed. |

# Reporting for specific materials, systems and methods

We require information from authors about some types of materials, experimental systems and methods used in many studies. Here, indicate whether each material, system or method listed is relevant to your study. If you are not sure if a list item applies to your research, read the appropriate section before selecting a response.

## Materials & experimental systems

| n/a | Involved in the study |
| --- | --- |
| ☒ | ☐ Antibodies |
| ☒ | ☐ Eukaryotic cell lines |
| ☒ | ☐ Palaeontology and archaeology |
| ☒ | ☐ Animals and other organisms |
| ☒ | ☐ Clinical data |
| ☒ | ☐ Dual use research of concern |

## Methods

| n/a | Involved in the study |
| --- | --- |
| ☒ | ☐ ChIP-seq |
| ☒ | ☐ Flow cytometry |
| ☒ | ☐ MRI-based neuroimaging |
